# Emodin inhibits viability, proliferation and promotes apoptosis of hypoxic human pulmonary artery smooth muscle cells via targeting miR-244-5p/DEGS1 axis

**DOI:** 10.1186/s12890-021-01616-1

**Published:** 2021-07-31

**Authors:** Li Yi, JunFang Liu, Ming Deng, Huihua Zuo, Mingyan Li

**Affiliations:** 1 Special Medical Service Center, Zhujiang Hospital, Southern Medical University, Guangzhou, 510282 China; 2 Pulmonary and Critical Care Medicine, Zhujiang Hospital of Southern Medical University, Guangzhou, 510282 China; 3 Department of Cardiology, Fuwai Hospital, Chinese Academy of Medical Sciences, NO.12, Langshan Road, Nanshan District, Shenzhen, 518057 Guangdong China; 4 Department of Cardiology, The Second Affiliated Hospital of Guangzhou Medical University, NO. 250 Changgangdong Road, Guangzhou, 510260 Guangdong China

**Keywords:** Emodin, Pulmonary arterial hypertension, Pulmonary artery smooth muscle cells, Proliferation, Apoptosis, miR-244-5p, DEGS1

## Abstract

**Objective:**

This study aimed to determine the effects of emodin on the viability, proliferation and apoptosis of human pulmonary artery smooth muscle cells (PASMCs) under hypoxia and to explore the underling molecular mechanisms.

**Methods:**

PASMCs were cultured in a hypoxic environment (1% oxygen) and then treated with emodin. Cell viability, proliferation and apoptosis were evaluated using CCK-8 assay, EdU staining assay, western blot and Mito-tracker red CMXRos and Annexin V-FITC apoptosis detection assay. The microRNA (miRNA)/mRNA and protein expression levels were assessed by quantitative real-time PCR and western blotting, respectively. Based on transcriptomics and proteomics were used to identify potential signaling pathways. Luciferase reporter assay was utilized to examine the interaction between miR-244-5p and DEGS1.

**Results:**

Emodin at 40 and 160 µM concentration-dependently suppressed cell viability, proliferation and migration, but enhanced cell apoptosis of PASMCs under hypoxia. Transcriptomic and proteomic analysis revealed that emodin could attenuate the activity of PI3K/Akt signaling in PASMCs under hypoxia. In addition, delta 4-desaturase, sphingolipid 1 (DEGS1) was found to be a direct target of miR-244-5p. Emodin could significantly up-regulated miR-244-5p expression and down-regulated DEGS1 expression in PASMCs under hypoxia. Furthermore, emodin-mediated effects on cell viability, migration, apoptosis and PI3K/Akt signaling activity of PASMCs under hypoxia were significantly attenuated by miR-244-5p knockdown.

**Conclusions:**

Our results indicated that emodin suppressed cell viability, proliferation and migration, promoted cell apoptosis of PASMCs under hypoxia via modulating miR-244-5p-mediated DEGS1/PI3K/Akt signaling pathway. MiR-244-5p/DEGS1 axis was initially investigated in this current study, which is expected to further the understanding of the etiology of pulmonary arterial hypertension.

**Supplementary Information:**

The online version contains supplementary material available at 10.1186/s12890-021-01616-1.

## Introduction

Patients with pulmonary arterial hypertension (PAH) mainly present with shortness of breath and progressive right heart failure. Multiple factors are involved in the etiology of this disease, including toxins and congenital cardiomyopathy [1]. Regardless of the causes, patients with PAH may suffer from hypoxia to some degree, particularly those who have underlying lung diseases [2]. Despite of the attempts in current pharmacotherapy which targets several signaling pathways including nitric oxide, endothelin and prostacyclin [3], PAH is a devastating and lethal cardio-pulmonary disease without effective treatment [4].

It has been revealed by histopathologic observations that in patients with PAH, the proliferation of pulmonary artery smooth muscle cells (PASMCs) were promoted, which were refractory to routine treatments [5]. The biological behaviors of these smooth muscle cells resemble those of cancerous cells in terms of increased Warburg metabolism, mitochondrial dynamics, and calcium-sensing receptor (CaSR) mediated cellular proliferation [6]. In addition, studies have demonstrated that protein expression of hypoxia-inducible factors (HIF) including HIF-1α and HIF-2α in PASMCs were up-regulated in PAH patients [7–9]. The hyperproliferation of PASMCs is recognized as a promising target for intervention in PAH-related vascular remodeling, therefore, it is of great scientific significance to explore the molecular mechanisms underlying PASMC hyperproliferation, in order to develop potential new therapies for PAH treatment.

Emodin, a chemical compound, of the anthraquinone family, is extracted from *polygonum cuspidatum* and *polygonum multiflorum* [10]. Studies have revealed that emodin exerted the inhibitory effects on cell survival and cycle progression [11–13]. Moreover, emodin could suppress the proliferation of aortic vascular smooth muscle cells by regulating microRNAs (miRNAs) expression [14]. The phosphatidylinositol 3-kinase (PI3K)/Akt (PI3K/Akt) signaling pathway is a classical signaling pathway in cells, and has always been a focus of interest in various diseases including cancers [15], diabetes [16], PAH [17], due to its role in cell growth, cell proliferation, cell migration and deregulated apoptosis. Previous studies also emphasized the effects of PI3K/Akt on survival of PASMCs [18] and the Warburg effects in PASMCs [19]. All the above evidence reveals the adverse effects of the PI3K/Akt pathway on PAH treatment. Therefore, adjusting PI3K/Akt may be an effective strategy for vascular remodeling in PAH [20]. It is noteworthy that emodin could suppress the activity of PI3K/Akt signaling [21–23]. These findings suggested that potential ability of emodin in regulating PAH via PI3K/ Akt pathway.

MicroRNAs (miRNAs) are small (~ 22 nucleotide long) non-coding RNAs that negatively regulate gene expression at the posttranscriptional level [24]. Previous studies showed that miRNAs also regulated the PAH pathogenesis, and could be regarded as biomarkers and therapeutic targets of PAH [25, 26]. The related miRNAs involved in PAH include miR-143 [27], miR-124 [28], miR-138, miR-25 [29], and so son. It is also indicated that miRNAs can regulated PAH via PI3K/Akt pathway [30], as well as the role of emodin in adjusting miRNAs [31].

In the present study, we for the first time demonstrated that emodin exerted the potential therapeutic efficiency on PAH, by inducing cellular apoptosis and inhibiting the viability proliferation of PASMCs under hypoxia condition. This effect was achieved by up-regulating miR-224-5p. This study indicated that treatment with emodin may achieve therapeutic effect by targeting the miR-244-5p/delta 4-desaturase, sphingolipid 1 (DEGS1) axis, so as to inhibit cell viability and proliferation, and promote cell apoptosis of PASMCs, therefore introducing a new potential approach to treat PAH.

## Materials and methods

### Cell culture

Human PASMCs were obtained from the American Type Culture Collection (ATCC, Manassas, USA). Cells were cultured in Dulbecco’s modified Eagle medium/Ham’s F-12 medium (DMEM/F-12, Thermo Fischer Scientific, Waltham, USA) supplemented with 10 % fetal bovine serum (FBS; Gibco, Thermo Fischer Scientific). The cells were cultured in a 5 % CO_2_ incubator at 37 °C.

### Drug treatment and cell transfections

Human PASMCs were exposed to hypoxia conditions (1% O_2_, 5% CO_2_, 94% N_2_) [32, 33] or normoxic condition (21% O_2_, 5% CO_2_, 74% N_2_) for 24 h. Other experiments were performed after corresponding treatments. Emodin (purity ≥ 98.0%) was purchased from MedChemExpress (Shanghai, China). Emodin was dissolved at a concentration of 320 mM in dimethyl sulfoxide (DMSO, purity ≥ 99.5.0%, Sigma) as stock solution, stored at − 20 °C. Stock solutions of emodin in DMSO were diluted into the corresponding working solution using cell culture medium before each experiment, and the final concentration of DMSO was less than 0.1%. Human PASMCs were pre-treated with emodin solution at different concentrations (0, 10, 40 and 160 µM) for 1 h, and then incubated in hypoxia condition or normoxic condition for 24 h, respectively (Additional file 1: Figure. S1).

The miR-224-5p mimic, miR-224-5p mimic negative control (NC) (NC mimics), miR-224-5p inhibitor and miR-224-5p inhibitor negative control (NC inhibitor) were purchased from GenePharma (Suzhou, China). Small hairpin RNA (shRNA) silencing DEGS1 (DEGS1 shRNA), the negative control shRNA (sh-NC), DEGS1 overexpressing vector (pcDNA3.1-DEGS1) and pcDNA3.1 were obtained from GenePharma. For the cell transfections, 1 × 10^6^ cells were cultured in 24-well plates with 1 mL complete medium for 24 h until they were 90% confluence, and cells were transfected using Lipofectamine 3000 reagents (Invitrogen, Carlsbad, USA) according to the manufacturer’s protocol.

### Quantitative real-time PCR (qRT-PCR)

Total RNA was isolated from the cells by using Trizol reagent (TaKaRa, Japan). MicroRNAs were extracted using Molpure Cell/Tissue miRNA Kit (Yeasen, Shanghai, China). The mRNA was reversely transcribed using SuperScript IV Reverse Transcriptase kit (Invitrogen), and miRNAs were reversely transcribed using TaqMan MicroRNA Reverse Transcription Kit (Invitrogen). The mRNA and miRNA expression levels of related genes were measured by using SYBR Premix Ex TaqII kit (Tli RNaseH Plus) (, Japan) on in an ABI PRISM® 7900HT System (TaKaRa). U6 small nucleolar RNA was used as an endogenous control for miR-244-5p detection, while β-actin was used as the endogenous control to detect other mRNA expression levels. The primer sequences of these genes used in this study were listed in Table 1.

**Table 1:** The sequences of primers used for RT-qPCR

Gene	Primer sequences 5’→3’
MiR-224-5p	F: GCGCGCAAGTCACTAGTGG
	R:GTCGTATCCAGTGCAGGGTCCGAGGT ATTCGCACTGGATACGACAACGGAA
DEGS1	F: GCATCTTTACTTGGCCTGGGTT
	R: ACTCCAGCACCATCTCTCCTT
β-actin	F: CATGTACGTTGCTATCCAGGC
	R: CTCCTTAATGTCACGCACGAT
U6	F: CTCGCTTCGGCAGCACATATACT
	R: ACGCTTCACGAATTTGCGTGTC

### Western blotting assay

Cells were lysed with radio immune-precipitation assay (RIPA) lysis buffer and extraction buffer containing protease inhibitors (Sigma-Aldrich, USA) at 4 °C, and the concentrations of extracted protein samples were determined by BCA protein assay kit (Sigma-Aldrich) [34]. Equal amounts of protein samples (40 µg) were loaded on 10% sodium dodecyl sulfate-polyacrylamide gel electrophoresis (SDS-PAGE) gel (Beyotechnology, Shanghai, China) and resolved by gel electrophoresis. The resolved proteins were then transferred onto polyvinylidene fluoride (PVDF) membranes (Millipore, USA) followed by incubating with 2% bovine serum albumin at room temperature for 1 h. After that, the membranes were incubated with primary antibodies including anti-cleaved-caspase-3 (1:1000 dilution; #9662 Cell Signaling Technology, USA), anti-Ki-67 (1:1000 dilution, #ab15580, Abcam, UK), anti-Akt (1:1000 dilution, #4685, Cell Signaling Technology, USA), anti-phospho-Akt (Ser473) (1:1000 dilution, #4060, Cell Signaling Technology), anti-PI3K-gamma (1:1000 dilution, # ab32089, Abcam), anti-phospho-PI3K (1:1000 dilution, #4228, Cell Signaling Technology), anti-DEGS1 (1:1000 dilution, #ab124798, Abcam), anti-PNCA (1:1000 dilution, #ab29, Abcam), anti-Bax (1:1000 dilution, #ab32503, Abcam), anti-Bcl-2 (1:1,000 dilution, # ab32124, Abcam, UK) and anti-β-actin (1:1000 dilution, #ab8226, Abcam) at 4 °C overnight. On the next day, these membranes were incubated with the horseradish conjugated secondary antibodies at room temperature for 1 h. The protein bands were visualized using High-sig ECL Western Blotting Substrate (Tenon, Shanghai, China). β-actin was used as the internal control (Additional file 2).

### Cell viability and proliferation analysis

Cell viability and proliferation was measured by using Cell Counting Kit-8 (CCK-8) assay and EdU staining assay, respectively [35]. For CCK-8 assay, cells with different treatments were seeded into the 96-well plates at a density of approximately 4 × 10^4^ cells/well. Next, 10 µL of CCK-8 solution (MedChemExpress, USA) was added into each well and then were incubated at 37 °C for 1 h, then the absorbance of each sample was determined at 450 nm by using a microplate reader (Thermo Fisher Scientific). EdU staining assay was performed using BeyoClick™ EdU-488 Cell proliferation kit (Beyotime, China) according to the manufacturer’s instructions. Briefly, cells with different treatments were incubated with Edu working solution for 2 h and then fixed with 4 % paraformaldehyde (PFA) for 15 min at room temperature. After permeabilized with 0.3 % TritonX-100 for 15mim at room temperature, the cells cultured with 500 µL Click Additive Solution for 30mim at darkness. Subsequently, cell nucleus was counterstained with DAPI for 10mim. Finally, the EdU-positive cells were then observed under the fluorescence microscope, and the percentage of EdU-positive cells was calculated as the cell proliferation rate.

### Mitochondrial membrane potential and cell apoptosis assay

Cellular apoptosis was detected using Mitochondrial Membrane Potential and Apoptosis Detection Kit with Mito-Tracker Red CMXRos and Annexin V-FITC Apoptosis Detection Kit (Beyotechnology, Shanghai, China). Briefly, 2 µL of Mito-Tracker Red CMXRos solution and 5 µL of Annexin V-FITC solution were added into each well of 6-well plates and the plates were shaken gently. After that, the samples were incubated at 24 °C for 30 min. Then, the human PASMCs were visualized by fluorescence microscope (Nikon’s MicroscopyU, Tokyo, Japan). Living cells were negative for green fluorescence, while positive for red fluorescence. The apoptotic cells were positive in green fluorescence, while the red fluorescence was significantly reduced or negative.

### Wound healing assay

Wound healing assay was performed to assess cell migration [36]. After the cells received different treatments for 24 h, the culture medium was removed and phosphate buffer saline (Sigma-Aldrich, USA) was used to wash the cells, which was followed by scratch wound assay through applying a scratch on the plates with a sterile pipette. Then, the photographs of the wounds were taken immediately at 48 h by light microscope (Nikon), respectively. The closure area of wound was calculated as follows: migration area (%) = (A_0_ − A_n_)/A_0_ × 100, where A_0_ represents the area of initial wound area, and A represents the remaining area of wound at the metering point.

### Transcriptome sequencing and analysis

After RNA was extracted by utilizing Trizol reagent, transcriptome sequencing was carried out using the method as previously reported [37]. Genes with a false-discovery rate (FDR) value of < 0.05 and fold change value > 2 were defined as differentially expressed genes (DEGs). DEGs were further analyzed by exploring the network of Kyoto Encyclopedia of Genes and Genomes (KEGG, https://www.kegg.jp/).

### Proteomics and bioinformatic analysis

Proteins were obtained by using RIPA reagent mixed with protease inhibitor cocktail at 4 °C for 30 min. After that, the cell lysates were harvested, digested and fractionated as previously described [38]. Raw data was acquired and analyzed using the proteomics data analysis platform of MaxQuant (v1.6.17.0, Max-Planck-Institute of Biochemistry, Germany) for genes with FDR value of < 0.05 and the absolute value of fold change of > 2 [39]. In this way, DEGs were identified, and then the functional enrichment analysis of DEGs was performed on KEGG.

### Cell transfection and dual luciferase reporter assays

The binding sites between miR-224-5p and DEGS1 3’ untranslated region (3’UTR) were predicted by TargetScan. The DEGS1 3’UTR was amplified from genomic DNA and was subcloned into the pGL3 luciferase reporter vector (Promega, Madison, USA) to construct the wild type (WT) luciferase reporter vector, DEGS1-3’UTR-WT. The mutant (MUT) luciferase reporter vector, DEGS1-3’UTR-MUT, was generated by Site-Directed Mutagenesis kit (Thermo Fisher Scientific). For the luciferase reporter assay, human PASMCs were cultured in 96-well plates. When the cell confluence reached approximately 60 %, the cells were co-transfected with DEGS1-3’UTR-WT (or DEGS1-3’UTR-MUT), miR-224-5p mimic, miR-224-5p mimic NC, miR-224-5p inhibitor and miR-224-5p inhibitor negative control (NC inhibitor), respectively. After 48 h, the luciferase activity was measured by Dual-Luciferase Reporter Assay kit (Promega) according to the manufacturer’s instruction.

### Statistical analysis

All the data analysis was performed using GraphPad Prism 8.0 (GraphPad Software, La Jolla, USA), and the data were presented as mean ± standard deviation. The statistical significance between comparisons was evaluated using Student’s t-test or one‑way analysis of variance followed by Dunnett’s multiple comparison tests. *P* < 0.05 was considered statistically significant.

## Results

### Emodin inhibited the viability, proliferation and promoted apoptosis of PASMCs under hypoxia

**Fig. 1:**
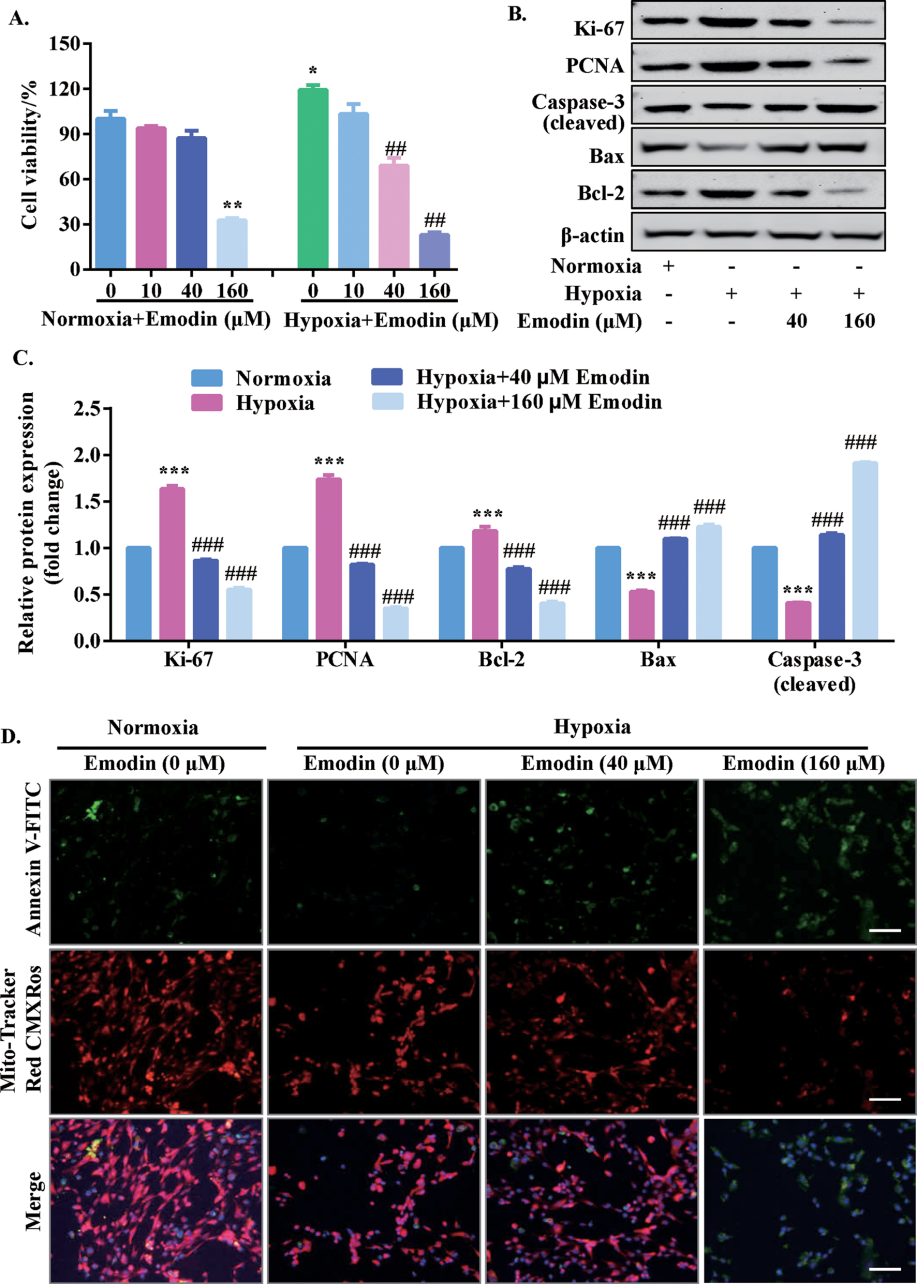
Emodin inhibited cell viability and promoted apoptosis in human PASMCs under hypoxia. **a** CCK-8 assay was performed to determine PASMC’s viability after the cells with emodin at different concentrations (0, 10, 40 and 160 µM) for 24 h. **b**, **c** Typical western blotting and quantitative analysis of Ki-67, PNCA, Bax, Bcl-2 and cleaved caspase-3 in PASMCs. **d** Assays of Mitochondrial Membrane Potential and Apoptosis Detection Kit with Mito-Tracker Red CMXRos and Annexin V-FITC was performed to determine cellular apoptosis; scale bar = 100 μm. N = 3. Dunnett’s multiple comparisons test, *P < 0.05 and **P < 0.01 versus normoxia but 0 µM emodin group; ^#^*P* < 0.05 and ^##^*P* < 0.01 versus hypoxia but 0 µM emodin group

**Fig. 2:**
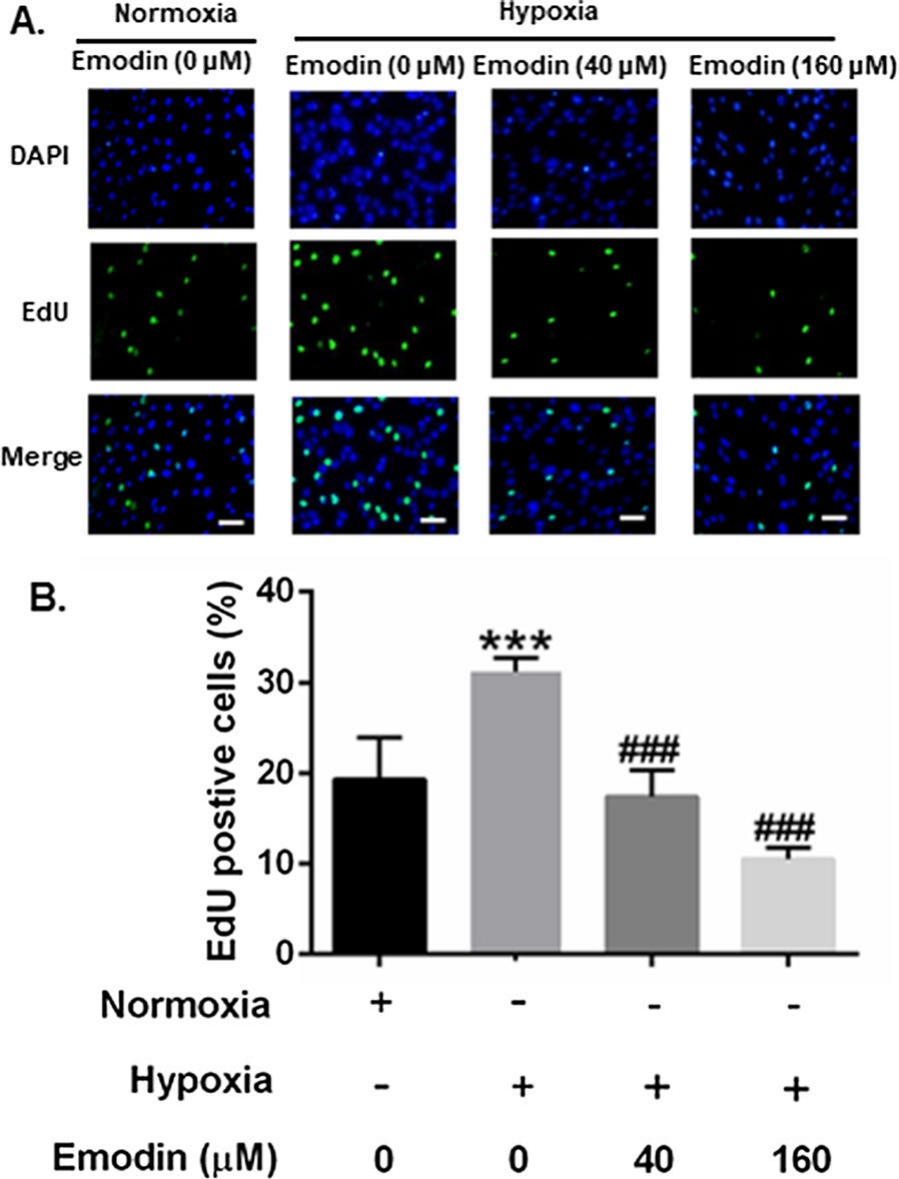
Emodin inhibited human PASMC proliferation under hypoxia. **a** Emodin’s effect of reducing hypoxia-treated PASMC proliferation as assessed by EdU staining assay; scale bar = 50 μm. **b** Quantitative analysis of proliferation properties. N = 3. Dunnett’s multiple comparisons test, **P < 0.001 versus normoxia but 0 µM emodin group; ^###^*P* < 0.001 versus hypoxia but 0 µM emodin group

As shown in Additional file 1: Fig. S2, the CCK-8 assay results demonstrated that hypoxia treatment increased the cell viability of PASMCs notably at 24 h when compared with the normoxic treatment. In the setting of hypoxia, 40 µM of emodin decreased cell viability considerably, while in normoxic condition, it had no effects on cell viability and LDH release of PASMCs (Fig. 1 a and Additional file 1: Fig. S3-S4). To further confirm the inhibitory effect emodin on PASMCs proliferation, EdU staining assay and western blot assay were performed to detect cell proliferation. The results of EdU staining assay showed that the hypoxia treatment significantly increased the percentage of EdU-positive cells than normoxia treatment, while emodin treatment could significantly reduce the percentage of EdU-positive cells in hypoxic condition (Fig. 2). The results of western blot revealed that hypoxia up-regulated the protein expression levels of Ki-67 and proliferative cell nuclear antigen (PNCA), (Fig. 1b, c), which was significantly attenuated by the treatment with emodin (40 and 160 µM). Moreover, protein expression levels of cleaved caspase-3, Bax and Bcl-2 were measured by western blot assay. The results showed that hypoxia up-regulated the protein expression levels of Bcl-2 but decreased the protein levels of Bax and cleaved caspase-3 (Fig. 1b, c), which was significantly attenuated by the treatment with emodin (40 and 160 µM). In addition, the PASMCs were stained with Mito-Tracker Red CMXRos and Annexin V-FITC to detect cellular apoptosis (Fig. 1d). Compared with the normoxia group, the hypoxia/emodin (40 µM) co-treatment group showed a considerable increase in apoptosis. In contrast, 40 µM emodin failed to enhance cell apoptosis of PASMCs under normoxic condition (Additional file 1: Fig. S5). Therefore, 40 µM of emodin were used for further research. Besides, we also evaluated the effects of emodin on pulmonary artery endothelial cells (HPAECs) and the cell viability was determined. Emodin at 40 µM had no effects on cell viability of HPAECs (Additional file 1: Fig. S6). The above results indicated that under hypoxia, 40 µM emodin could suppress the viability, proliferation and promote apoptosis of PASMC under hypoxic conditions.

### Emodin inhibited the migration of PASMC under hypoxia

**Fig. 3:**
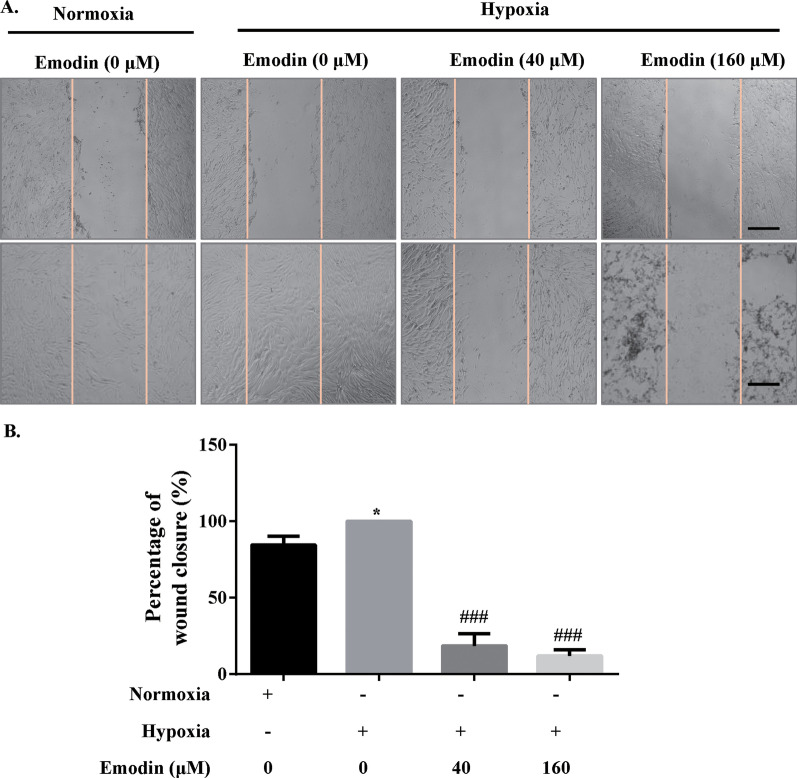
Emodin inhibited human PASMC migration under hypoxia. **a** Emodin’s effect of reducing hypoxia-treated PASMC migration as assessed by wound healing assay; scale bar = 100 μm. **b** Quantitative analysis of migration properties. N = 3. Dunnett’s multiple comparisons test, *P < 0.05 and **P < 0.01 versus normoxia but 0 µM emodin group; ^#^*P* < 0.05 and ^##^*P* < 0.01 versus hypoxia but 0 µM emodin group

The migratory potential of PASMCs was assessed by wound healing assay. Emodin at 40 and 160 µM inhibited the migration of PASMCs under hypoxia (Fig. 3a, b). It was also noteworthy that cell migration of PASMCs was slightly increased after hypoxia treatment (Fig. 3b). Therefore, the results indicated that emodin exerted inhibitory effects on the migration of PASMCs migration in the setting of hypoxia.

### Emodin targeted PI3K/AKT signaling pathway under hypoxia

**Fig. 4:**
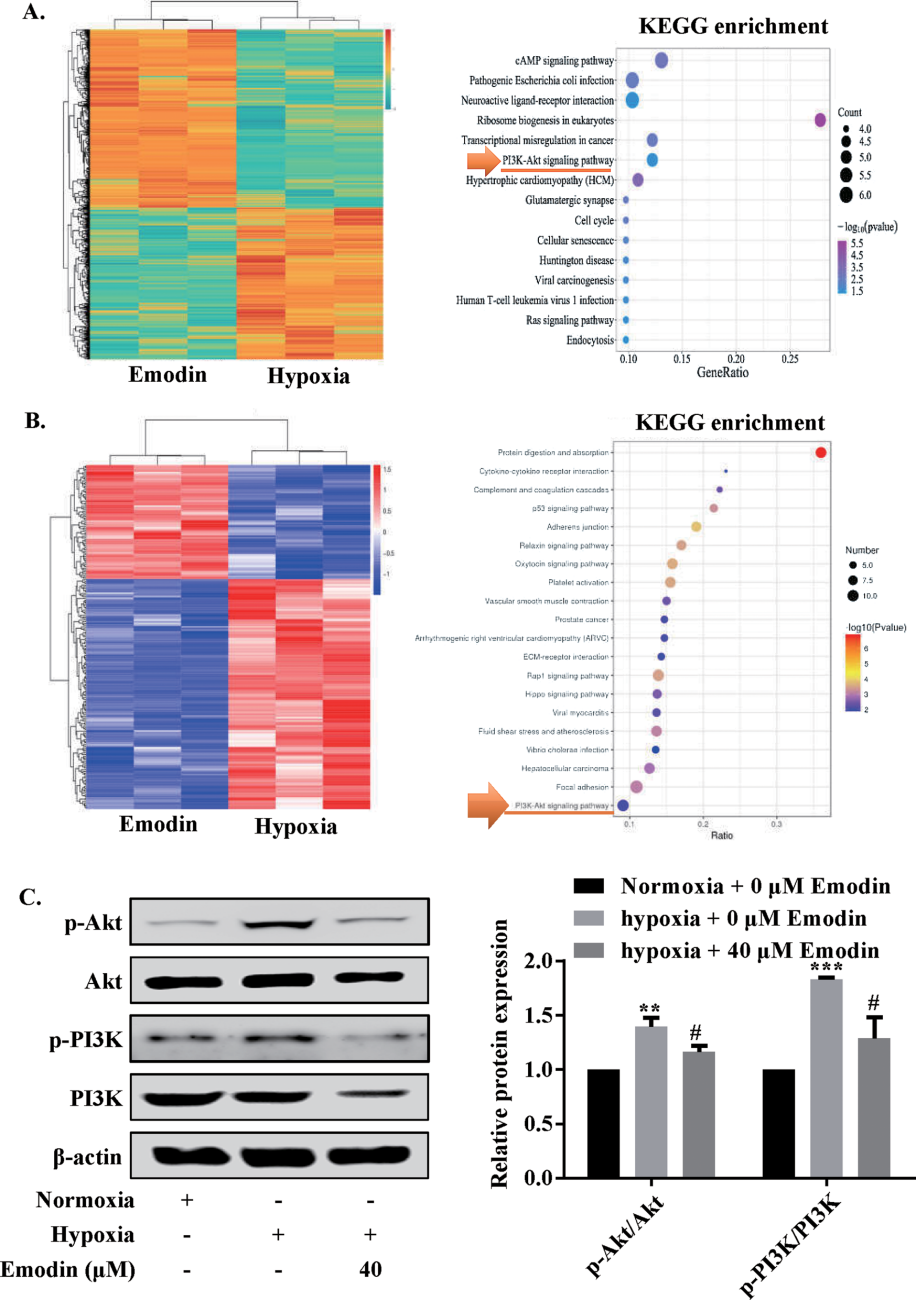
Emodin inhibited PI3K/Akt signaling pathway in human PASMCs under hypoxia exposure. **a** Transcriptomic analysis of human PASMCs co-treated with 40 µM emodin and hypoxia. The left panels show the heatmap displaying a subset of differentially expressed genes of PASMCs treated with emodin for 24 h (absolute FC > 2, *P* < 0.05) and the right panels show that the most enriched pathways of differentially expressed genes. **b** Proteomic analysis of human PASMCs co-treated with 40 µM emodin and hypoxia. The left panels show the heatmap displaying a subset of differentially expressed proteins of human PASMCs treated with emodin for 24 h (absolute FC value > 1, *P* < 0.05) and the right panels show the most enriched pathway of proteins differentially expressed. **c** Western blotting and quantitative analysis of p-PI3K/PI3K ratio and p-Akt/Akt ratio. N = 3. Dunnett’s multiple comparisons test, β-actin was used as the internal control. *P < 0.05 and **P < 0.01 versus normoxia but 0 µM emodin group; ^#^*P* < 0.05 and ^##^*P* < 0.01 versus hypoxia but 0 µM emodin group

Transcriptomic analysis was performed after human PASMCs were treated with 40 µM emodin and hypoxia for 24 h, in order unveil the underlying mechanism. The heatmap of enriched KEGG pathways of DEGs were displayed in Fig. 4 a, and the DEGs were enriched in the PI3K/Akt signaling pathway. To confirm this, proteomic analysis was carried out, and the results suggested that PI3K/Akt signaling pathway was activated when these cells were subjected to both emodin and hypoxia treatments (Fig. 4b). The western blot assay showed that under the condition of hypoxia, the ratios of p-Akt/Akt and p-PI3K/PI3K were higher when compared to those of the normoxia group, which was partially reversed by emodin treatment (Fig. 4c). Collectively, these resulted implied that emodin inhibited the activation of PI3K/Akt signaling pathway in PASMCs under hypoxia.

### Emodin downregulated DEGS1 expression in PASMCs under hypoxia exposure

**Fig. 5:**
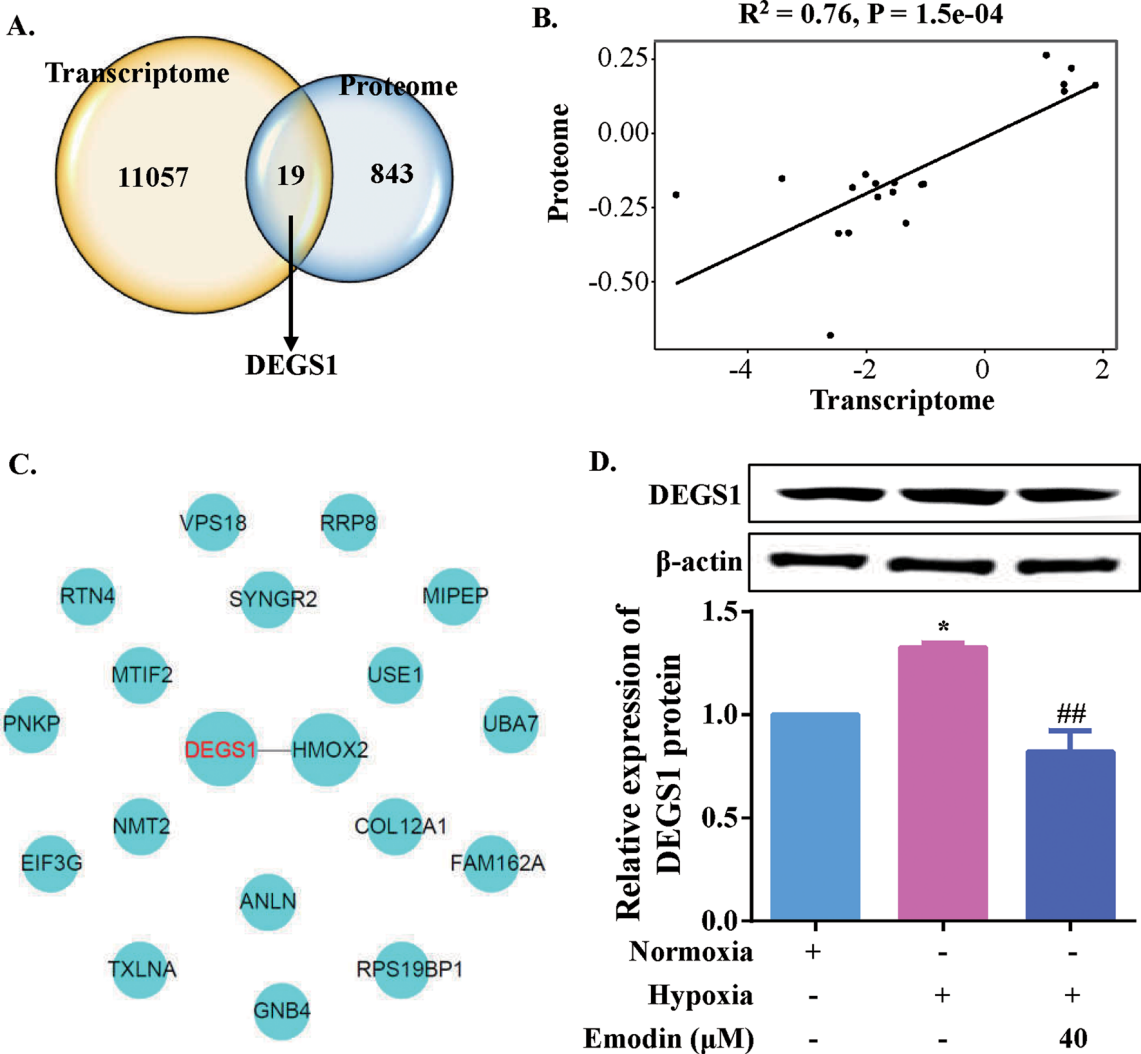
Emodin downregulated DEGS1 expression in human PASMCs under hypoxia. **a** Comparison and integration analysis of differential transcriptomic and proteomic analysis. Venn diagram revealed 19 genes expressed differentially at both mRNA and protein levels. **b** Analysis of Pearson correlation coefficient showed that differential expression of these 19 genes was significantly correlated (R = 0.76, *P* < 0.001). **c** Differential expression of these 19 genes were used to construct PPI network to screen key genes. **d** Western blots and quantitative analysis of DEGS1. N = 3. Dunnett’s multiple comparisons test, *P < 0.05 and **P < 0.01 versus normoxia but 0 µM emodin group; ^#^*P* < 0.05 and ^##^*P* < 0.01 versus hypoxia but 0 µM emodin group

Differential transcriptomic and proteomic analysis were compared and integrated, in order to further understand the mechanisms of action of emodin in regulating the PI3K/Akt signaling pathway. As shown in Fig. 5a, the Venn diagram identified common 19 DEGs between transcriptome and proteome analysis. There was a linear correlation between the differential expression of these 19 genes (Fig. 5b). Furthermore, a protein-protein interaction (PPI) network of these 19 genes was built, and a link between DEGS1 and heme oxygenase 2 (HMOX2) was revealed (Fig. 5c). To validate this finding, protein expression level of DEGS1 was measured (Fig. 5d). DEGS1 has been found to be essential to the synthesis of lipids. In this study, it was discovered that protein expression level of DEGS1 was upregulated in PAMSCs subjected to hypoxia, in contrast to the normoxia group (Fig. 5d). It is worth mentioning that in the setting of hypoxia, the protein expression level of DEGS1 decreased after PASMCs were treated with 40 µM emodin, as compared to the hypoxia group. Taken together, it was concluded that emodin inhibited the activity of PI3K/Akt pathway in PASMCs possibly via targeting DEGS1.

### MiR-244-5p could target DEGS1 in PASMCs

**Fig. 6:**
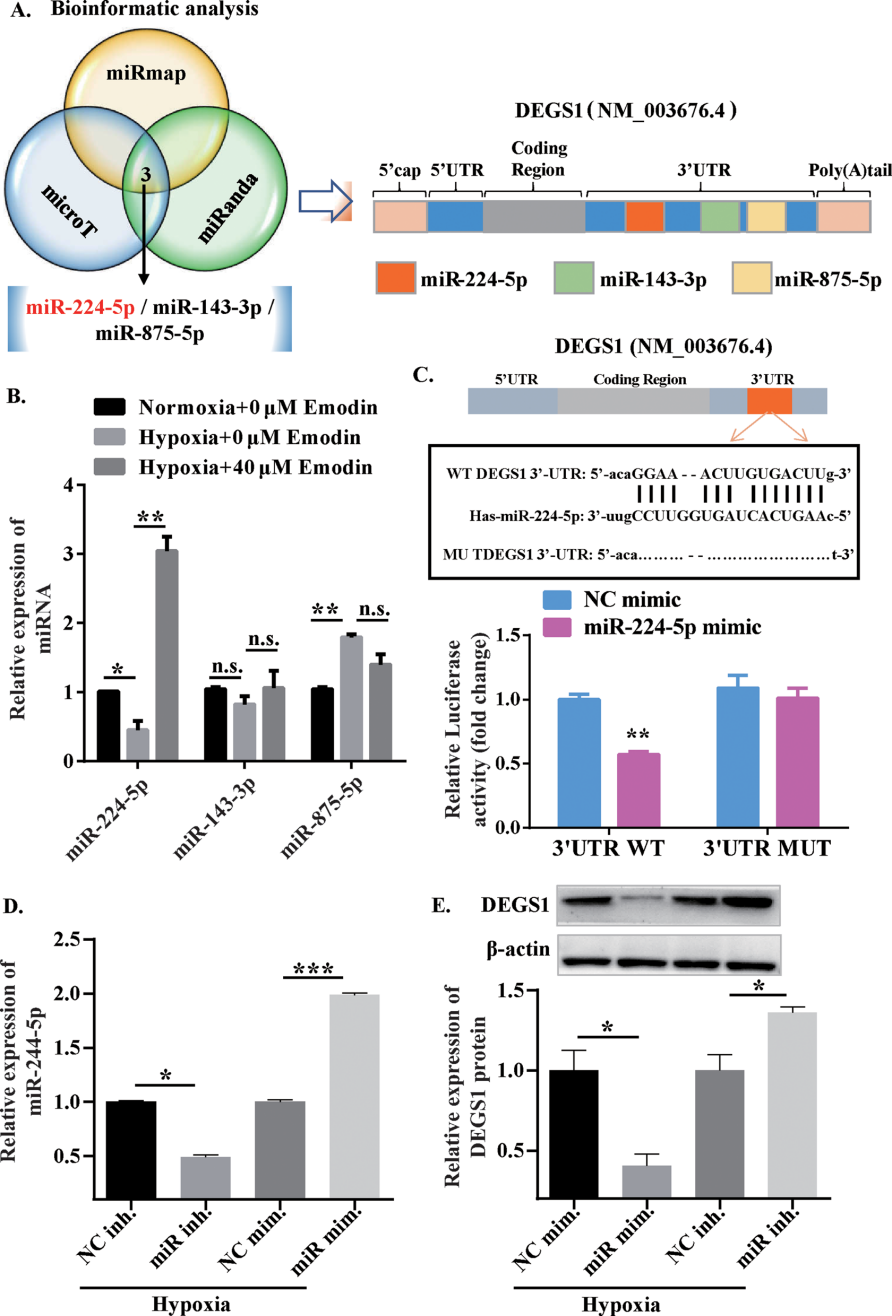
DEGS1 is a direct target of miR-244-5p human PASMCs. **a** Bioinformatic analysis was used to predict the potential miRNAs targeting DEGS1. **b** Human PASMCs were treated with emodin for 24 h, and then the relative expression levels of miR-244-5p, miR-143-3p and miR-875-5p were detected using RT-qPCR. **c** Schematic diagram of a predicted binding site of miR-244-5p in the 3’UTR-wild type of DEGS1 mRNA (3’UTR-WT) and the 3’UTR-mutant of DEGS1 mRNA (3’UTR-MUT); the luciferase activity was determined using the dual luciferase reporter system. **d** The expression of miR-224-5p were determined by RT-qPCR in PASMCs transfected with miR-224-5p mimics or inhibitor. **e** The protein expression of DEGS1 were determined by western blot in PASMCs transfected with miR-224-5p mimics or inhibitor. N = 3. **P < 0.01 versus negative control (NC) mimic groups

Based on the exploration of the microRNA databases (miRmap, microT and miRanda), three miRNAs, namely the miR-244-5p, miR-143-3p and miR-875-5p were predicted to interact with DEGS1, and their correlations with DEGS1 were displayed in Fig. 6a. Subsequently, expression levels of these three miRNAs were quantified by qRT-PCR. As shown in Fig. 6b, the expression level of miR-244-5p in the hypoxia and emodin (40 µM) co-treatment group was the highest as compared to the other two miRNAs. Therefore, it was speculated that miR-244-5p could be a potential target of DEGS1. To verify this assumption, the luciferase reporter constructs, namely the DEGS1-3’UTR-WT and DEGS1-3’UTR-MUT, were generated. In the wild-type group, miR-224-5p mimic and its negative control were used to transfect PASMCs respectively, and the luciferase activities were measured. As shown in Fig. 6c, the luciferase activity of the miR-224-5p mimic group was considerably decreased, in contrast to the negative control group. Meanwhile, PASMCs were co-transfected with DEGS1-3’UTR-MUT and miR-224-5p mimic or miR-224-5p negative control, respectively, followed by measurement of luciferase activity. In the mutant luciferase reporter constructs, the luciferase activity of the miR-224-5p mimic group was almost equivalent to that of the negative control group. Moreover, the miR-224-5p mimic increased the expression of miR-224-5p, which could be inhibited by miR-224-5p inhibitor (Fig. 6d). The overexpression of miR-224-5p inhibited the protein expression of DEGS1, and miR-224-5p knockdown promoted the expression of DEGS1 (Fig. 6e). Based on these findings, it was assumed that DEGS1 was one of the direct target gene of miR-224-5p.

### Emodin decreased DEGS1 expression by upregulating miR-244-5p in PASMCs under hypoxia

**Fig. 7:**
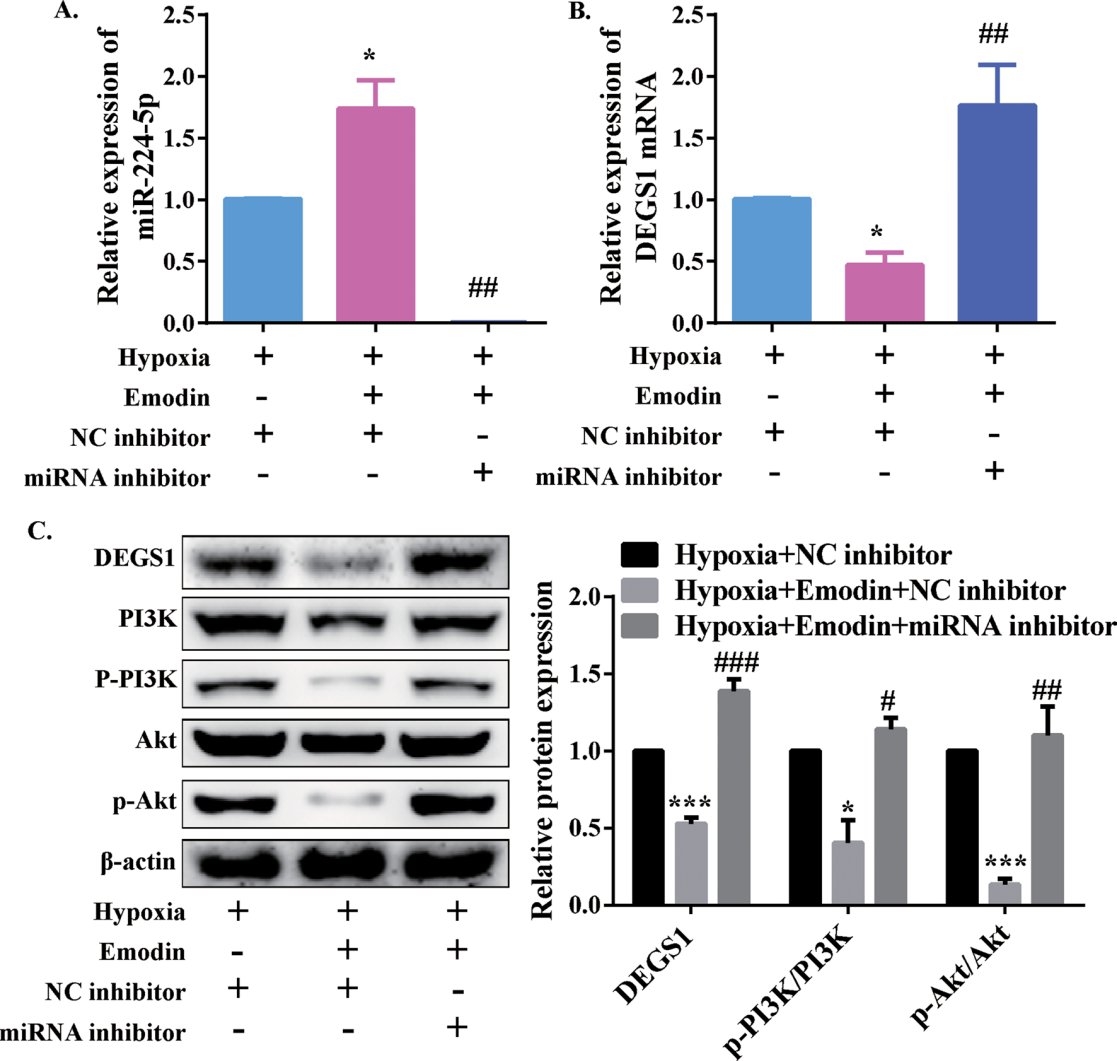
Emodin inhibited DEGS1 expression via upregulating miR-244-5p expression in human PASMCs under hypoxia. **a** The relative expression levels of miR-244-5p were detected by RT-qPCR. **b** The relative mRNA expression levels of DEGS1 were detected by RT-qPCR. **c** Typical western blotting and quantitative analysis of DEGS1, p-Akt, Akt, p-PI3K and PI3K normalized to β-actin. N = 3. *P < 0.05 and**P < 0.01 versus hypoxia + NC inhibitor group; ^#^*P* < 0.05 and ^##^*P* < 0.01 versus hypoxia + emodin + NC inhibitor group

To determine whether miR-224-5p was involved in emodin-induced DEGS1 downregulation, PASMCs were cultured in a hypoxic environment and treated with 40 µM emodin, miR-244-5p inhibitor and the negative control, respectively, followed by measurement of the expression of miR-244-5p. In the hypoxia and emodin co-treatment group, the miR-244-5p expression level was increased moderately, compared with the hypoxia control group (Fig. 7a). Next, the mRNA expression level of DEGS1 was quantified by RT-qPCR. In the setting of hypoxia, treatment of PASMCs with emodin resulted in a substantial decrease in the mRNA expression level of DEGS1, whereas cells in the emodin and miR-244-5p inhibitor co-treatment group showed an increase in mRNA expression level of DEGS1 (Fig. 7b). Furthermore, the interaction between DEGS1 and PI3K/Akt signaling pathway was explored at a protein level. In Fig. 6c, it was shown that under hypoxia, PASMCs treated with emodin led to decreased expression level of DEGS1 and the activity of PI3K/Akt signaling pathway was downregulated significantly, as compared with the hypoxia control group. However, in a hypoxic environment, the addition of emodin to PASMCs transfected with miR-244-5p inhibitor upregulated DEGS1 expression and activated PI3K/Akt signaling pathway, as compared to the hypoxia and emodin co-treatment group. We also determined whether DEGS1 regulated cell viability, apoptosis and migration. As shown in Additional file 1: Fig. S7A-S7D, the PASMCs with overexpression of DEGS1 showed higher ability of viability and migration, and lower expression of cleavage caspase 3. Overexpression of DEGS1 also promoted PI3K/Akt activation (Additional file 1: Fig. S7E). However, after knockdown of DEGS1, these phenomena above were inhibited. These results indicated that in a hypoxic environment, emodin could upregulate miR-244-5p expression, which in turn inhibited DEGS1 expression. And then, the downregulated DEGS1 suppressed cell viability, promoted cell apoptosis, and inhibited cell migration, as well as decreased PI3K/Akt activation in PASMCs.

### Emodin suppressed PASMC’s viability and induced apoptosis by upregulating miR-244-5p/DEGS1 axis

**Fig. 8:**
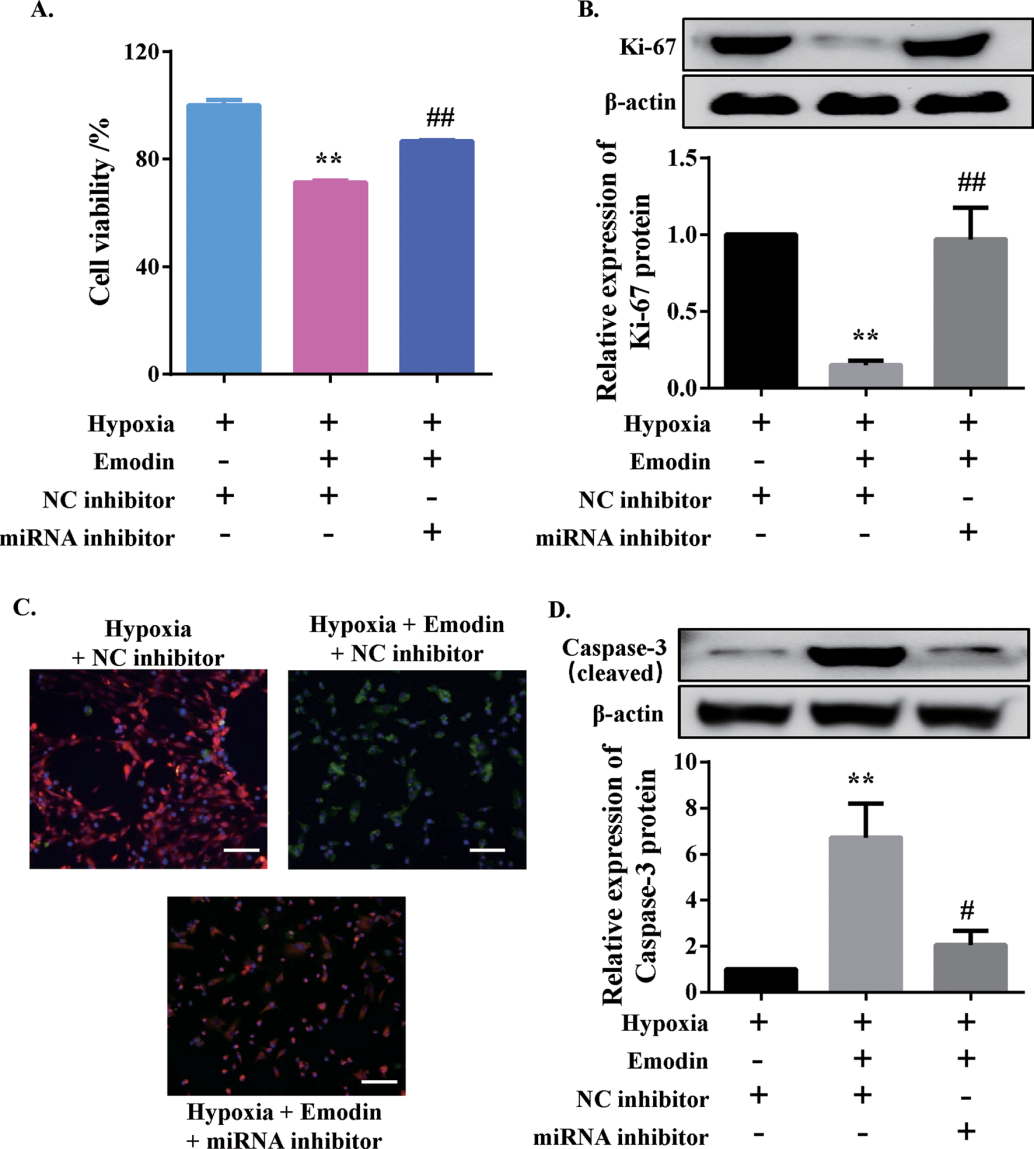
Emodin regulated human PASMC viability and apoptosis under hypoxia exposure by upregulating miR-244-5p. **a** CCK-8 assay was performed to determine human PASMC viability. **b** Typical western blotting and quantitative analysis for Ki-67 protein normalized to β-actin. **c** The Mitochondrial Membrane Potential and Apoptosis Detection Kit with Mito-Tracker Red CMXRos and Annexin V-FITC assay was performed to determine apoptosis of human PASMCs; scale bar = 100 μm. **d** Typical western blotting and quantitative analysis of cleaved caspase-3 normalized to β-actin. N = 3. *P < 0.05 and **P < 0.01 versus hypoxia + NC inhibitor group; ^#^*P* < 0.05 and ^##^*P* < 0.01 versus hypoxia + emodin + NC inhibitor group

After treatment of emodin at 40 µM, a moderate decrease in cellular activity of hypoxic PASMCs (Fig. 8a) and protein expression level of Ki-67 (Fig. 8b) were observed. Yet, in a hypoxic environment, emodin treatment of PASMCs that were transfected with miR-244-5p inhibitor increased cell viability (Fig. 8a) and upregulated Ki-67 protein expression level (Fig. 8b), as compared to the emodin/hypoxia co-treatment group. To determine apoptosis, cells were stained with Mito-Tracker Red CMXRos and Annexin V-FITC dyes (Fig. 8c) and the protein expression level of cleaved caspase-3 was measured (Fig. 8d). The results showed that in the setting of hypoxia, silencing miR-244-5p in emodin-treated HPASMCs reduced apoptosis rate, in contrast to the hypoxia/emodin co-treatment group. We also validated whether DEGS1/PI3K/Akt axis was involved in the effects of emodin. Over expression of DEGS1 reversed the effects of emodin on cell viability, apoptosis and migration (Additional file 1: Fig. S8A-S8C). Similarly, after activation of PI3K/Akt signaling by 740Y-P, the effects of emodin were also blocked (Additional file 1: Fig. S8D-S8F). Based on these findings, it was concluded that emodin could suppress the viability of PASMCs and induce cellular apoptosis via upregulating miR-244-5p/DEGS1/PI3K/Akt axis.

## Discussion

Emodin has been reported to be effective in treating various diseases such as malignant diseases and severe acute respiratory syndrome [13]. Previous studies had seldom explored whether this Chinese herb could be effective against PAH. In this current study, we found that under a hypoxic condition, emodin exhibited inhibitory effects on the migration of PASMCs as well as induced cellular apoptosis. Based on the results of bioinformatics analysis, it was discovered that emodin upregulated the expression of miR-244-5p under hypoxia and that miR-244-5p inhibited the activation of PI3K/Akt pathway via targeting and regulating the expression of DEGS1.

Numerous studies have revealed that hypoxia could accelerate the progression of PAH in different ways. For instance, hypoxia suppresses the activity of cyclin-dependent kinase inhibitors through upregulating miRNAs, thus allowing vascular smooth muscles to proliferate [40, 41], which may lead to decreased compliance of pulmonary arteries and aggravate right heart failure. In addition, hypoxia acts as a stimulus in mediating inflammation by increasing NF-κB activity, and the expression of various proinflammatory cytokines, particularly IL-6, will be increased [42]. Proinflammatory cytokines, such as IL-6, IL-1β and IL-33, can accelerate the proliferation of vascular smooth muscle cells [43–45]. Previous studies discover the important role of PI3K/Akt pathway in PAH pathology and targeting PI3K/Akt pathway can attenuates hypoxia-induced PAH efficiently. For example, the dioscin and baicalin, the natural compounds extracted from traditional Chinese medicine, can attenuates PAH via inhibiting PI3K/Akt [20, 46]. In this current study, emodin suppressed the activity of PI3K/Akt signaling pathway. It has been reported that hypoxia could activate this signaling pathway, and that the deposition of some extracellular matrices (ECMs), such as collagen I, collagen II and laminin, maintained the activation of this signaling pathway so as to promote the proliferation and migration of vascular smooth muscles [47]. Moreover, this pathway is associated with NF-κB signaling pathway to allow more calcium influx into cells, which results in progressive calcification of pulmonary arteries [48]. As emodin boasts the potential to downregulate PI3K/Akt signaling pathway, it is likely that these risks may be eliminated by this Chinese herb. In addition, it has been shown that emodin could alleviate inflammation via inhibiting the activity of NF-κB, thus it was considered as a novel adenosine monophosphate (AMP)-activated protein kinase (AMPK) activator which would exert protective effects on cardiovascular system [49–51]. Although a large number of literatures have demonstrated the important role of PI3K/Akt in PAH, as well as its correlation with other signaling pathways and inflammatory factors, these associations still need to be repeatedly verified in our future studies to confirm their specific role in emodin. Our results indicated that PI3K/Akt activation reversed the effects of emodin, suggesting that PI3K/Akt acted as the downstream factor of emodin.

Besides, our results also revealed the interaction between miRNA-mRNA and its role play in PASMCs under hypoxia after emodin treatment. Our results verified that miR-244-5p targeting DEGS1 and regulating PI3K/Akt. In the previous experiment, we screened miRNAs in PASMCs after emodin treatment, and found that miR-244-5p significantly increased. Although miRNAs usually have multiple targets, the downregulation of DEGS1 was particularly significant after the silencing of miR-244-5p. Based on these changes, our study demonstrated the key role of miR-244-5p/DEGS1 in inhibitory effects of emodin on PASMCs. In addition, DEGS1 has been found to be involved in the metabolism of sphingolipid and adipocyte differentiation [52, 53], be associated with many degenerative neurologic disorders such as leukodystrophy [54] and play a role in the self-renewal of hematopoietic stem cells [55]. However, studies on DEGS1 and miR-244-5p in PAH have rarely been reported. Therefore, our study might have found a new target for the treatment of PAH, but its specific mechanism needs to be further explored.

Our results showed that hypoxia treatment increased the cell viability of human PASMCs as determined by the CCK-8 assay, which was consistent with previous studies [56, 57]. Although 40 µM emodin suppressed the cell viability significantly in hypoxia condition, the cell viability of PASMCs in normoxic condition was stable after treatment with 40 µM emodin for 0, 12, 24 and 48 h. The LDH release and caspase activation were not induced by emodin in normoxic PASMCs. It suggested the low toxicity on normoxic PASMCs. On the other hand, it suggested that 40 µM emodin was effective in PASMCs while 160 µM was too high to induce cytotoxicity. Therefore, 40 µM emodin was more proper to be used. In addition, this study mainly investigated the effect of emodin on PAH model in vitro under hypoxic conditions, so no in-depth study was conducted on the toxicity of emodin in animals. The toxicity and protection of emodin will be fully considered in the follow-up work, so as to facilitate rational drug administration.

Despite of all the findings, inherent defects exist in this study. Firstly, the human PASMCs we used in the study may differ with those of patients diagnosed with PAH in terms of biologic behaviors. Rodent PAH models should have been constructed and the PASMCs should be harvested from these rodents for subsequent studies to improve the robustness of the study results. Secondly, although emodin has been proved to be effective against cancer, diabetes and inflammatory diseases, its adverse effects should not be neglected. For example, it may be toxic to liver, kidney and reproductive organs. Hence, how to eliminate these unfavorable side effects should become a major concern if emodin were to be applied in clinical practice. Thirdly, the current investigation is limited to the in vitro functional studies, and future in vivo studies are warranted to decipher the potential action of emodin in PAH.

## Conclusions

In conclusion, our results indicated that emodin suppressed cell viability, proliferation and migration, promoted cell apoptosis of PASMCs under hypoxia via modulating miR-244-5p-mediated DEGS1/PI3K/Akt signaling pathway. MiR-244-5p/DEGS1 axis was initially investigated in this current study, which is expected to further the understanding of the etiology of PAH.

## Supplementary Information

**Additional file 1**. Supplemental Figures for experimental results.

**Additional file 2**. Raw images for western blot.

## Data Availability

All the data are available upon reasonable request from the corresponding author (Huihua Zuo and Mingyan Li).
