# Supplementary material for: Emodin inhibits viability, proliferation and promotes apoptosis of hypoxic human pulmonary artery smooth muscle cells via targeting miR-244-5p/DEGS1 axis
Source: BMC Pulm Med. 2021 Jul 31;21:252. doi: 10.1186/s12890-021-01616-1 (PMC8325255; doi:10.1186/s12890-021-01616-1)
Supplement: Supplementary file 1 — Additional file 1. Supplemental Figures for experimental results. [file 12890_2021_1616_MOESM1_ESM.docx]

**Supplementary Fig. S1**

**
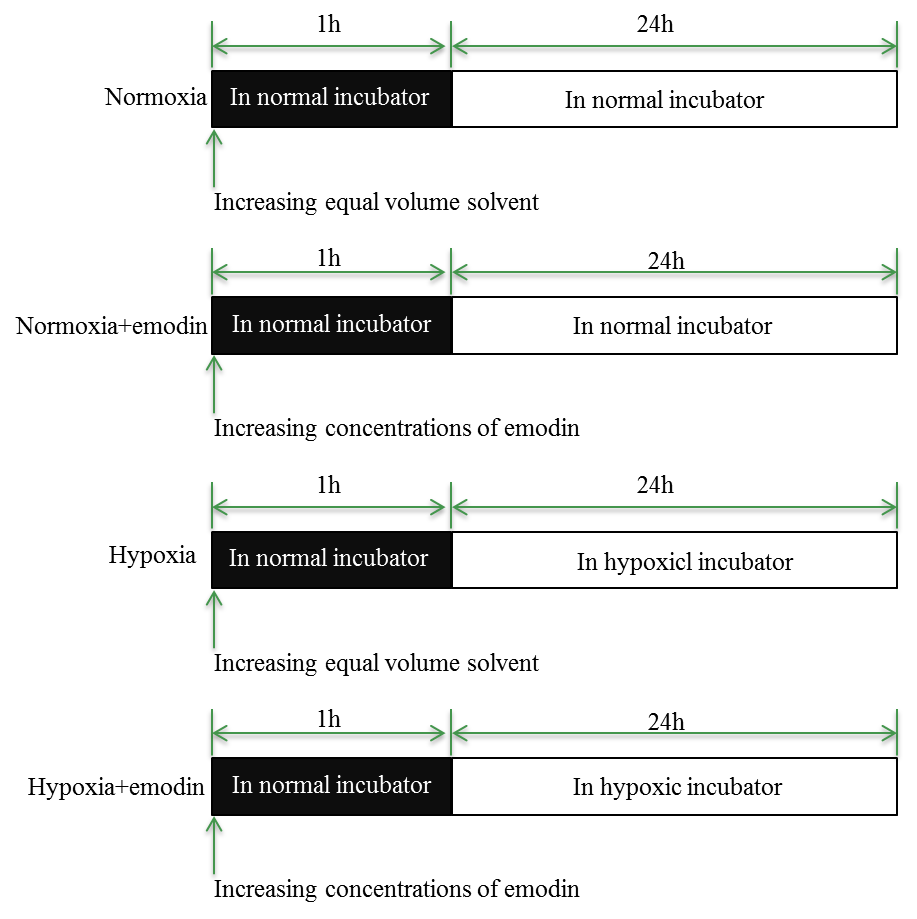
**

**Supplementary Fig. S1.** A diagram showing the experimental timeline of emodin treatment of human PASMCs in normal or hypoxic condition.

**Supplementary Fig. S2**

**
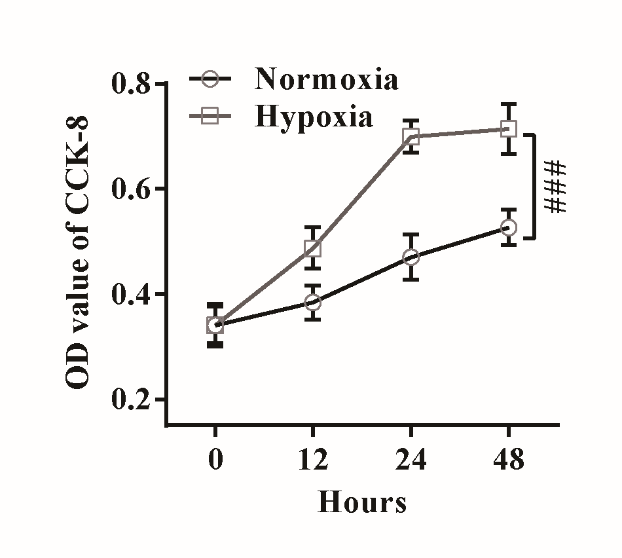
**

**Supplementary Fig. S2.** CCK-8 assay was performed to determine PASMC’s viability in normoxia or hypoxia condition for 12, 24 and 48 h, respectively. ^###^ *P*<0.001.

**Supplementary Fig. S3**


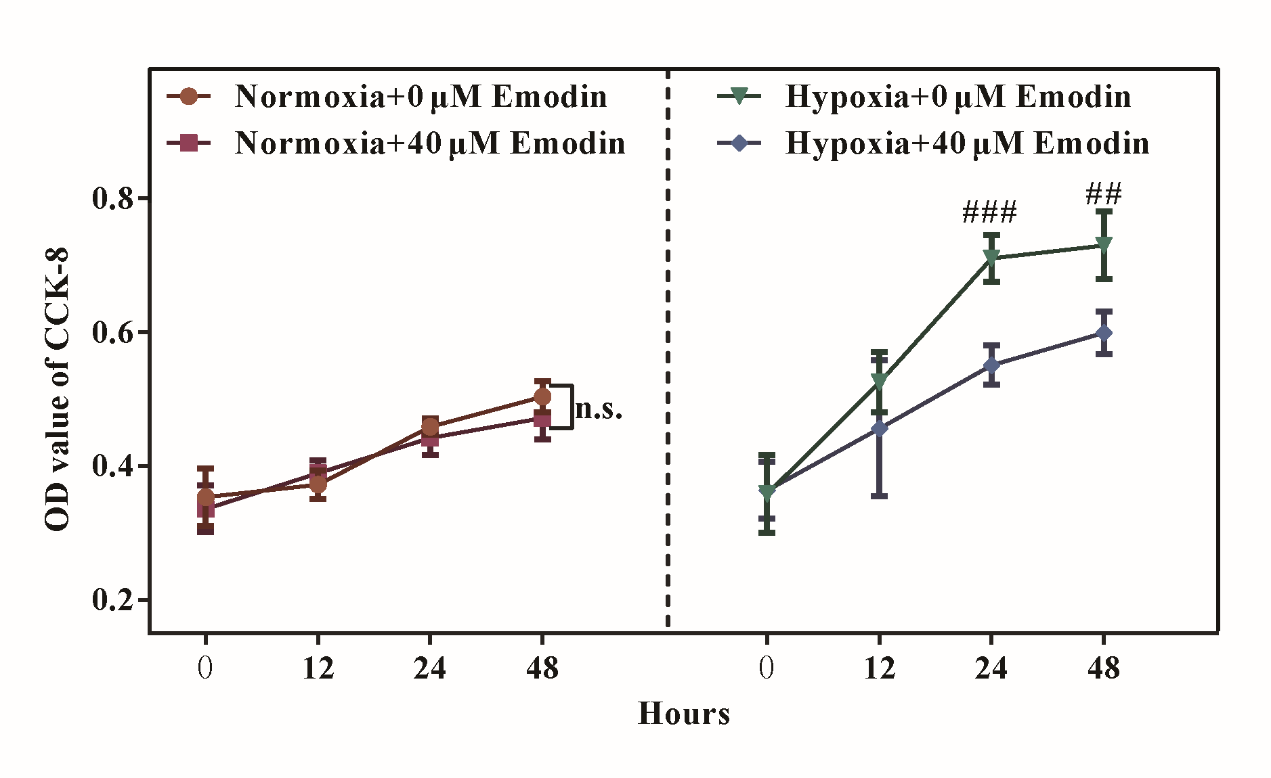


**Supplementary Fig. S3.** CCK-8 assay detected the effects of emodin at different concentrations on the viability of normoxic and hypoxic PASMCs. ^##^*P*<0.01, ^###^*P*<0.001 and ^n.s^*P*>0.05.

**Supplementary Fig. S4**

**
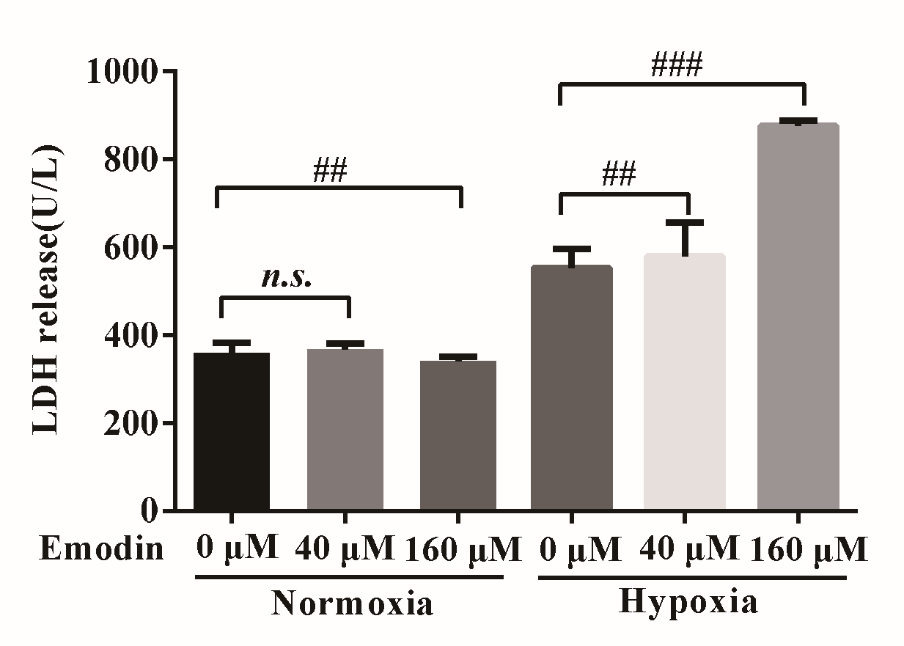
**

**Supplementary Fig. S4.** Effect of emodin at different doses on LDH release in normoxic and hypoxic PASMCs. ^##^*P*<0.01, ^###^*P*<0.001, ^n.s.^*P*>0.05

**Supplementary Fig. S5**


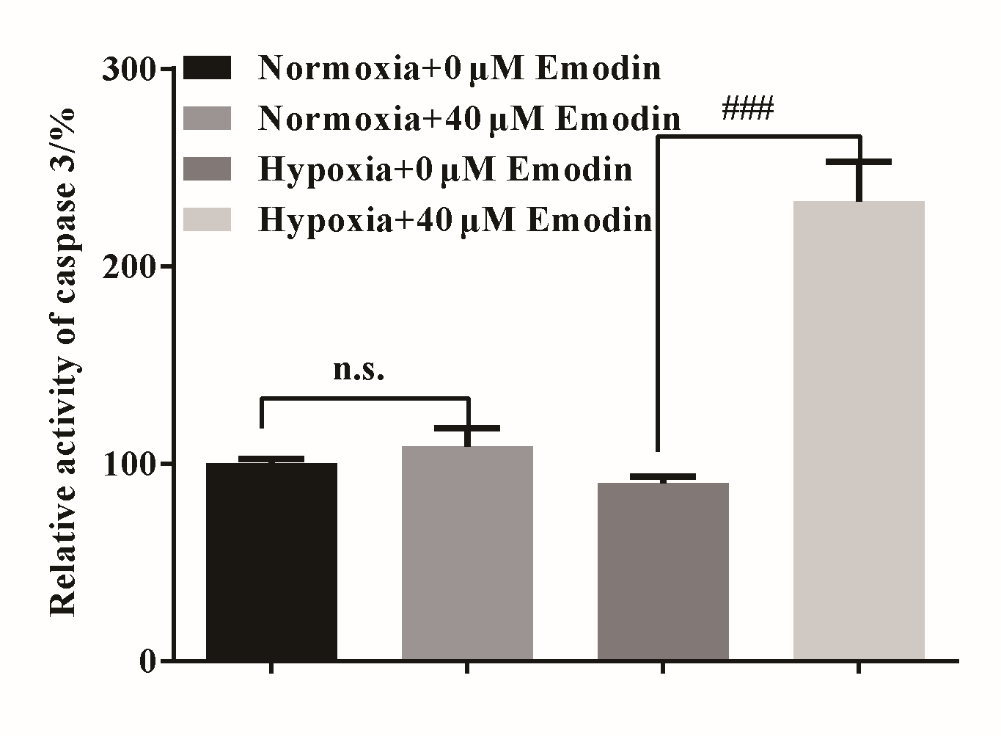


**Supplementary Fig. S5.** Effect of emodin on caspase-3 activity in normal and hypoxic PASMCs. ^###^ *P*<0.001, ^n.s.^ *P*>0.05.

**Supplementary Fig. S6**


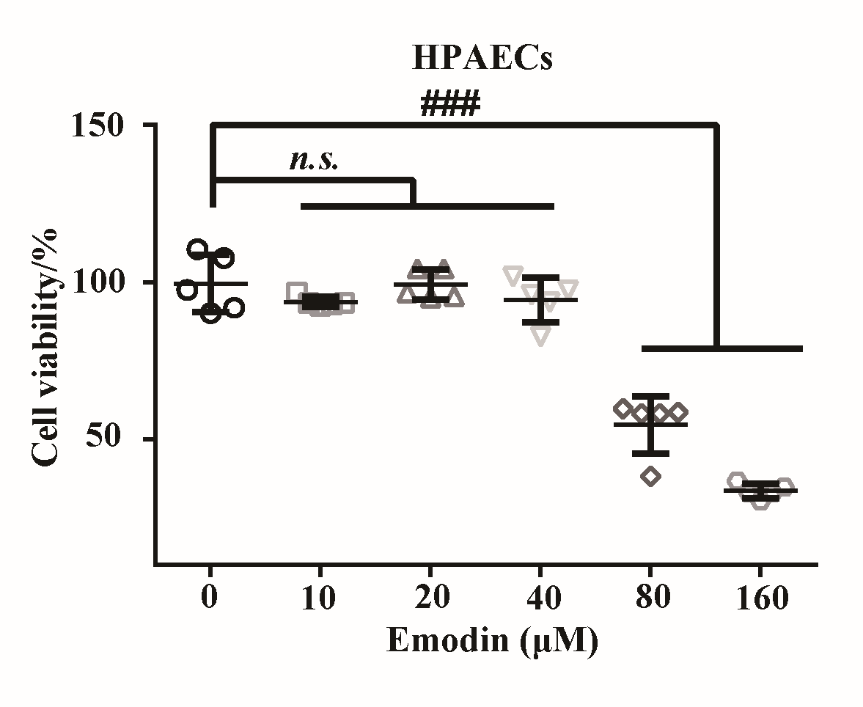


**Supplementary Fig. S6.** Effect of emodin on pulmonary artery endothelial cells (HPAECs). ^###^*P*<0.01, ^n.s.^ *P*>0.05

**Supplementary Fig. S7**

**
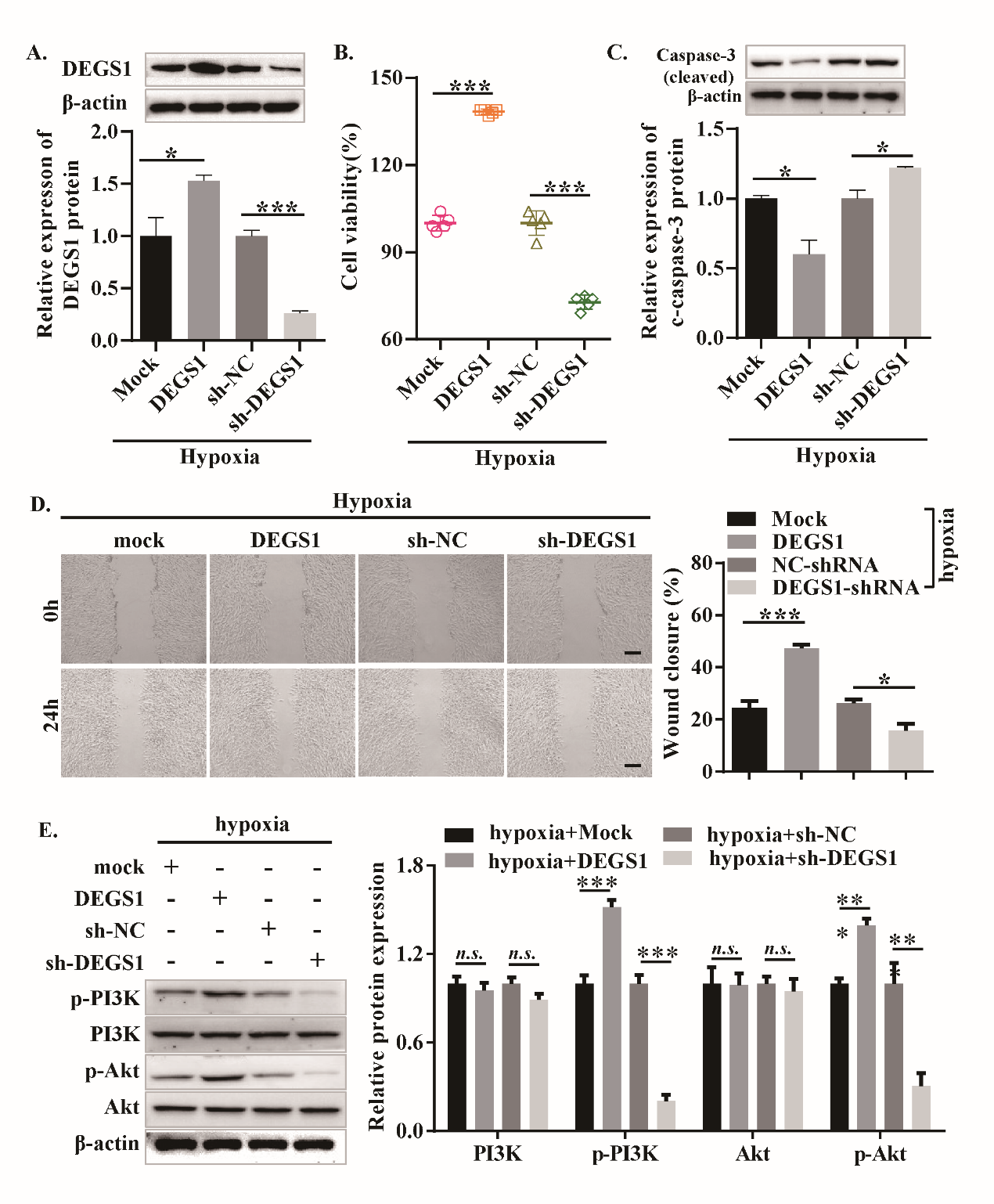
**

**Supplementary Fig. S7.** The effects of DEGS1 on the viability, apoptosis and migration of hypoxic pulmonary artery smooth muscle cells. (A) The DEGS1 protein expression were determined by western blot in PASMCs with overexpressed or knockdown of DEGS1 in hypoxia condition for 24h. (B) The cell viability was determined by CCK8 assay in PASMCs with overexpressed or knockdown of DEGS1 in hypoxia condition for 24h. (C) The cleavage-caspase 3 protein expression were determined by western blot in PASMCs with overexpressed or knockdown of DEGS1 in hypoxia condition for 24h. (D) The migration ability was determined by wound healing assay in PASMCs with overexpressed or knockdown of DEGS1 in hypoxia condition for 12h. (E) The PI3K/Akt signaling activation were determined by western blot in PASMCs with overexpressed or knockdown of DEGS1 in hypoxia condition for 24h. ^*^*P*<0.05, ^**^*P*<0.01, ^***^*P*<0.001,

**Supplementary Fig. S8**


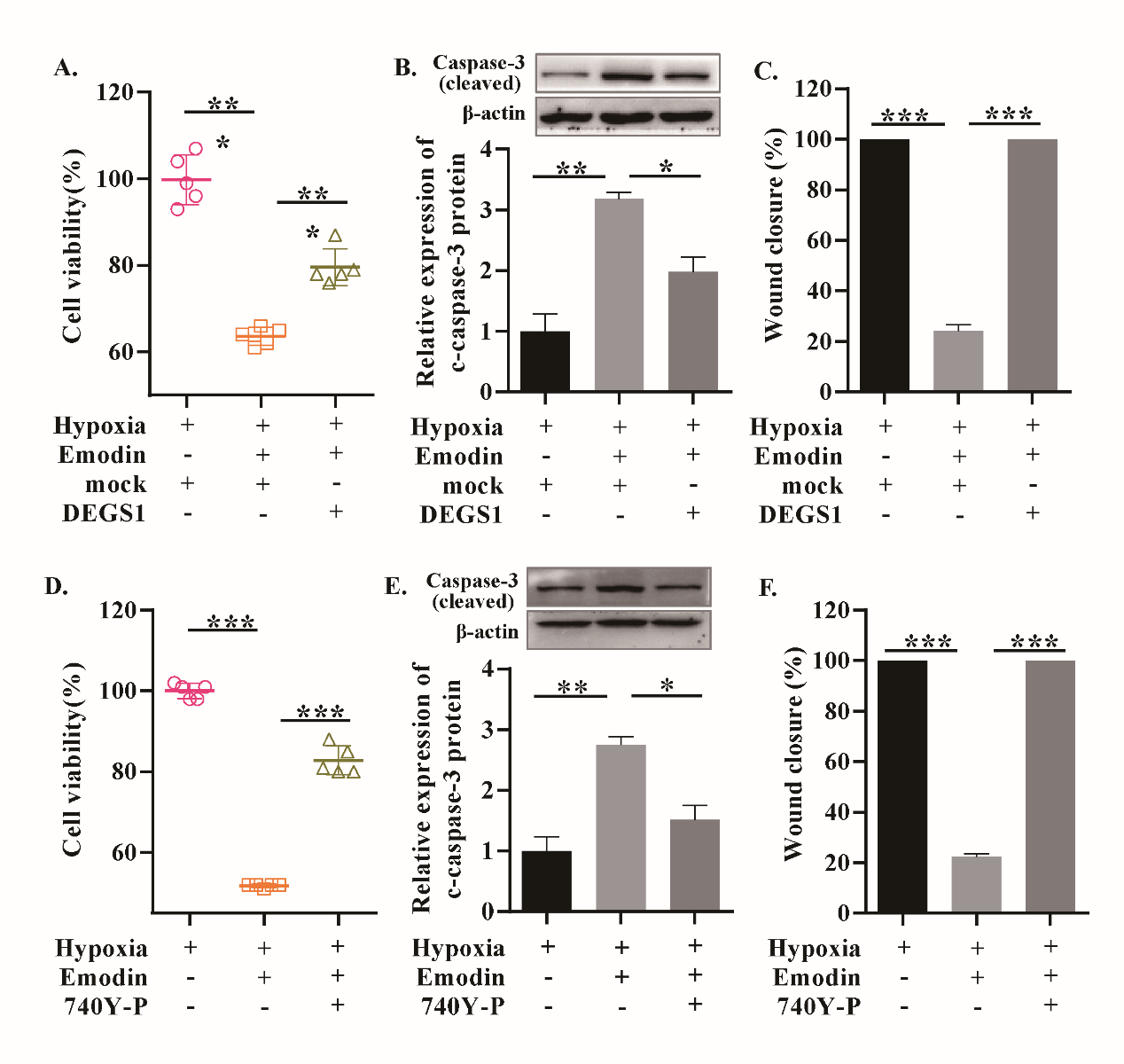


**Supplementary Fig. S8.** The regulatory effects of emodin on hypoxic PASMCs was achieved by DEGS1/PI3K/AKT axis. (A) The cell viability was determined by CCK8 assay. (B) The cleavage-caspase 3 protein expression were determined by western blot. (C) The migration ability was determined by wound healing assay. The PASMCs were treated with 40 μM emodin 740Y-P (PI3K agonist) in hypoxia condition. (D) The cell viability was determined by CCK8 assay. (E) The cleavage-caspase 3 protein expression were determined by western blot. (F) The migration ability was determined by wound healing assay. ^*^*p*<0.05, ^**^ *p*<0.01, ^***^ *p*<0.001.
