# Supplementary figures and images for: Emodin inhibits viability, proliferation and promotes apoptosis of hypoxic human pulmonary artery smooth muscle cells via targeting miR-244-5p/DEGS1 axis
Source: BMC Pulm Med. 2021 Jul 31;21:252. doi: 10.1186/s12890-021-01616-1 (PMC8325255; doi:10.1186/s12890-021-01616-1)

Figure S1

Ki-67  
395 kDa

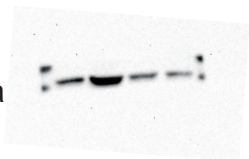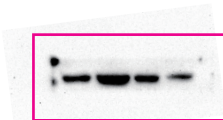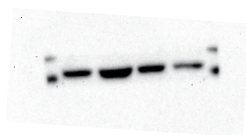

PCNA  
29 kDa

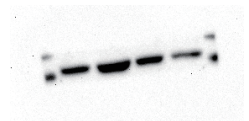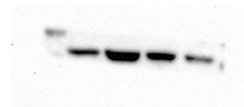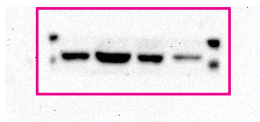

Caspase-3  
35 kDa

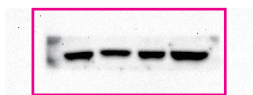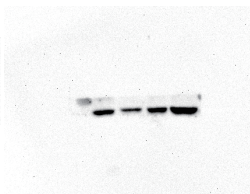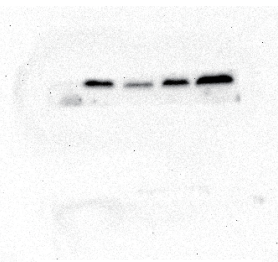

Bax  
21 kDa

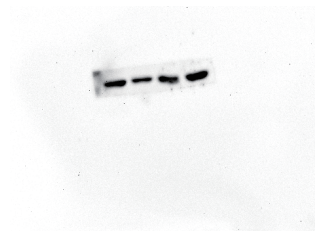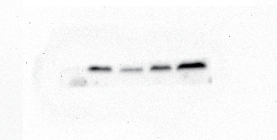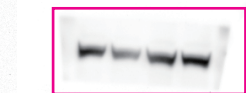

Bcl-2  
26 kDa

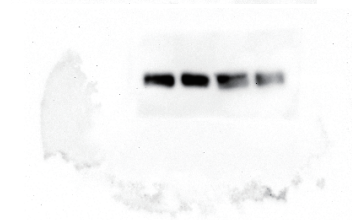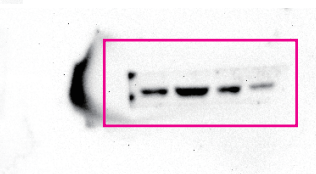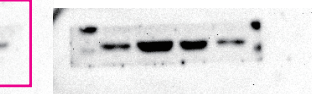

$\beta$ -actin  
42 kDa

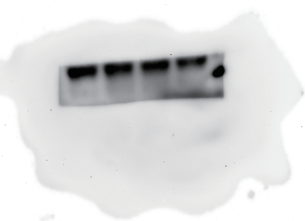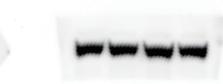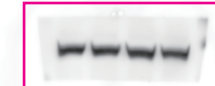

Supplement: Supplementary file 2 — Additional file 2. Raw images for western blot. [file 12890_2021_1616_MOESM2_ESM.zip › Western blot raw/FIGS1-FIG1B.pdf]

Figure S2

p-AKT  
56 kDa

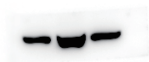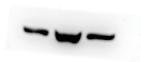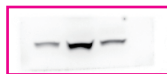

AKT  
56 kDa

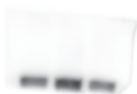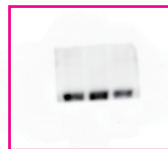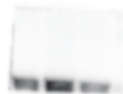

$\beta$ -actin  
42 kDa

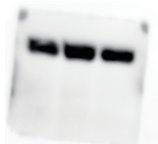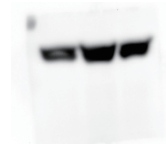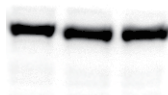

p-PI3K  
84 kDa

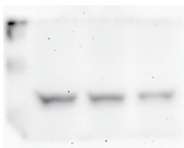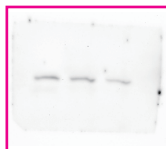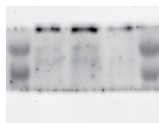

PI3K  
84 kDa

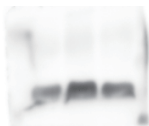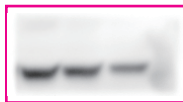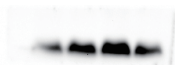

$\beta$ -actin  
42 kDa

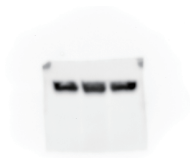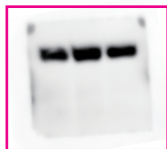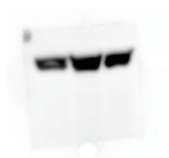

Supplement: Supplementary file 2 — Additional file 2. Raw images for western blot. [file 12890_2021_1616_MOESM2_ESM.zip › Western blot raw/FIGS2-FIG3C.pdf]

Figure S3

DEGS1  
38 kDa

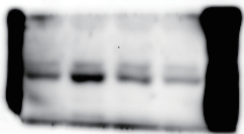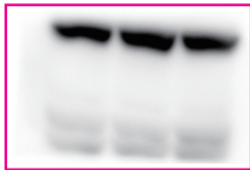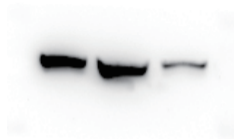

$\beta$ -actin  
42 kDa

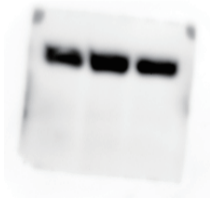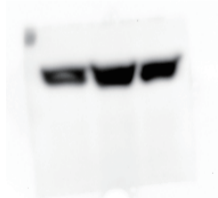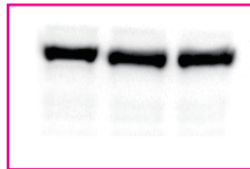

Supplement: Supplementary file 2 — Additional file 2. Raw images for western blot. [file 12890_2021_1616_MOESM2_ESM.zip › Western blot raw/FIGS3-FIG4D.pdf]

Figure S4

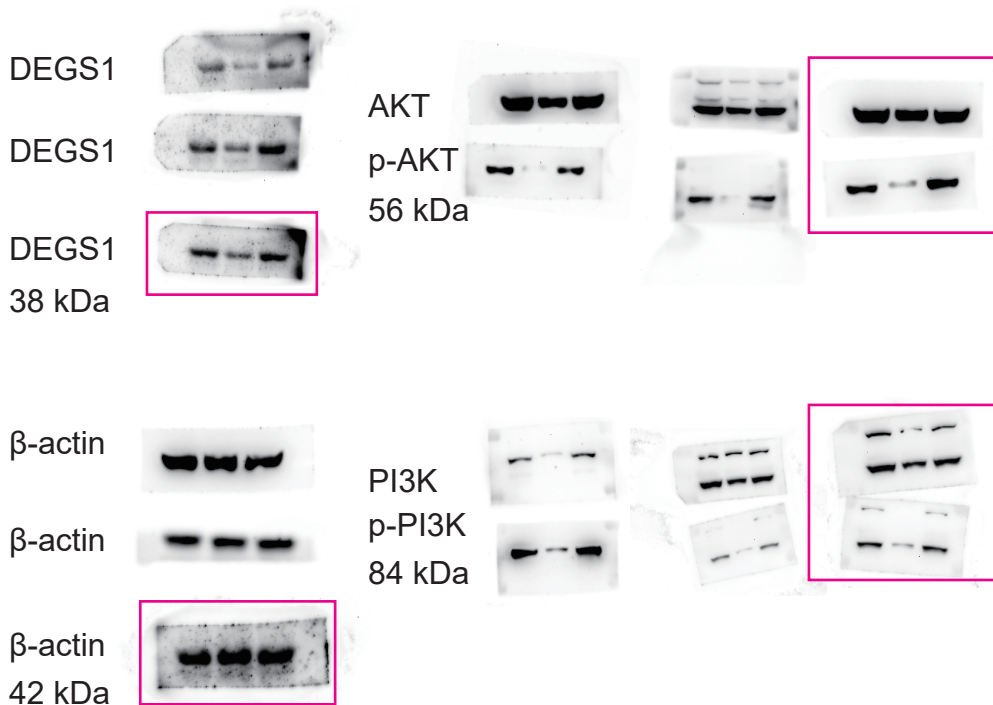

Supplement: Supplementary file 2 — Additional file 2. Raw images for western blot. [file 12890_2021_1616_MOESM2_ESM.zip › Western blot raw/FIGS4-FIG6C.pdf]

Figure S5

Ki-67  
395 kDa

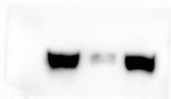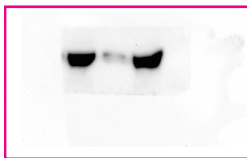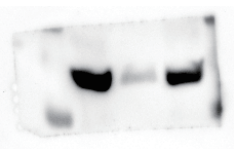

$\beta$ -actin  
42 kDa

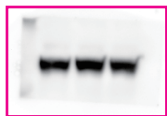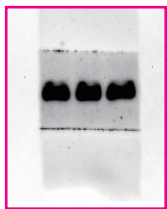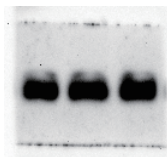

Caspase-3  
35 kDa

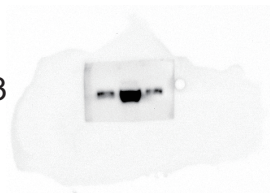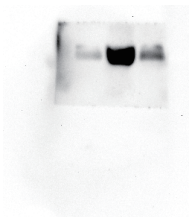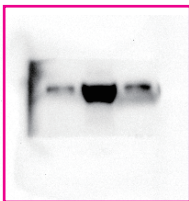

Supplement: Supplementary file 2 — Additional file 2. Raw images for western blot. [file 12890_2021_1616_MOESM2_ESM.zip › Western blot raw/FIGS5-FIG7BD.pdf]
